# A method to identify important dynamical states in boolean models of regulatory networks: application to regulation of stomata closure by ABA in *A. thaliana*

**DOI:** 10.1186/1471-2164-12-S4-S10

**Published:** 2011-12-22

**Authors:** Cristhian A Bugs, Giovani R Librelotto, José CM Mombach

**Affiliations:** 1 Departamento de Física, Universidade Federal de Santa Maria, Santa Maria (UFSM), RS, Brasil; 2 Universidade Federal do Pampa (UNIPAMPA), São Gabriel, RS, Brasil; 3 Departamento de Eletrônica e Computação, Universidade Federal de Santa Maria, Santa Maria (UFSM), Brasil

## Abstract

**Background:**

We introduce a method to analyze the states of regulatory Boolean models that identifies important network states and their biological influence on the global network dynamics. It consists in (1) finding the states of the network that are most frequently visited and (2) the identification of variable and frozen nodes of the network. The method, along with a simulation that includes random features, is applied to the study of stomata closure by abscisic acid (ABA) in *A. thaliana* proposed by Albert and coworkers.

**Results:**

We find that for the case of study, that the dynamics of wild and mutant networks have just two states that are highly visited in their space of states and about a third of all nodes of the wild network are variable while the rest remain frozen in True or False states. This high number of frozen elements explains the low cardinality of the space of states of the wild network. Similar results are observed in the mutant networks. The application of the method allowed us to explain how wild and mutants behave dynamically in the SS and determined an essential feature of the activation of the closure node (representing stomata closure), *i.e.* its synchronization with the AnionEm node (representing anion efflux at the plasma membrane). The dynamics of this synchronization explains the efficiency reached by the wild and each of the mutant networks.

**Conclusions:**

For the biological problem analyzed, our method allows determining how wild and mutant networks differ ‘phenotypically’. It shows that the different efficiencies of stomata closure reached among the simulated wild and mutant networks follow from a dynamical behavior of two nodes that are always synchronized. Additionally, we predict that the involvement of the anion efflux at the plasma membrane is crucial for the plant response to ABA.

**Availability:**

The algorithm used in the simulations is available upon request.

## Results

In plants transpiration occurs mainly at the leaves while their stomata open for the passage of CO_2_ and O_2_ during photosynthesis. This process is extremely costly for a plant, especially when the water supply is limited. The largest amount of water transpired by a higher plant is lost through the stomata that are controlled by surrounding guard cells that regulate the rate of transpiration. When guard cells become turgid they cause stomata to open allowing water to evaporate. When transpiration exceeds the absorption of water by the roots a loss of turgor occurs and the stomata close balancing the water lost and CO_2_ gain.

Sometimes severe environmental conditions, such as drought, low temperature, heat, high salinity or flooding, have adverse effects on plant growth and development. Plants respond to environmental stresses at cellular and molecular levels, as well as at physiological levels, so as to confer tolerance of the stress and ensure survival. During droughts conditions, the plant synthesizes a hormone called ABA which acts as a signal in a pathway that closes the plant stomata to reduce the water loss by transpiration [1].

**Figure 1:**
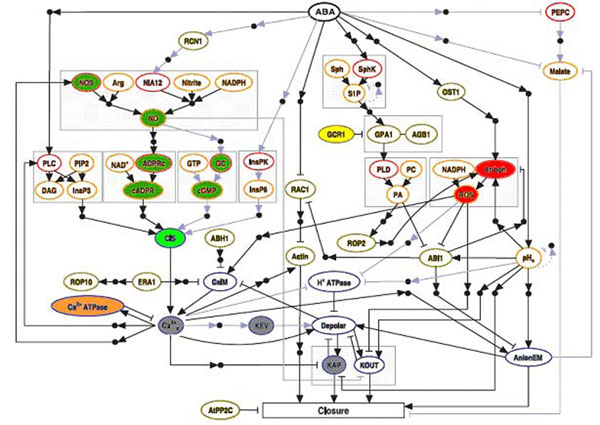
Adapted from [1]. Molecules including protein, hormones and second messengers in signal transduction pathways during ABA response. Small black filled circles represent putative intermediary nodes mediating indirect regulatory interactions. Arrowheads represent activation, and short perpendicular bars indicate inhibition. Light blue lines denote interactions derived from species other than Arabidopsis; dashed light-blue lines denote inferred negative feedback loops on pHc and S1P. Nodes involved in the same metabolic pathway or protein complex are bordered by a gray box; only those arrows that point into or out of the box signify information flow (signal transduction). The variable elements of the wild network are marked with colored ellipses and those with same color are in the same boolean State. The nodes that are held in a fixed state are marked with uncolored ellipses.

We apply our method (see section Methods) to a slightly modified version of the Boolean model of stomata closure regulation by ABA in *A. thaliana* proposed by Albert and coworkers [2]. They developed a model of the process of aperture and closure of the plant stomata induced by signal transduction via abscisic acid (ABA). The network simulation of the pathway proposed was validated and lately improved by the authors [1,2]. The network has 43 nodes and 69 interactions represented by links as seen in Fig. 1. Experimental information about the involvement of a specific component in ABA induced stomatal closure were used to build a network of interactions and many cellular components have been identified to function in ABA regulation of guard cell volume and thus of stomatal aperture. The interactions between these components govern many responses to various stimuli and plants respond to them in characteristic and generally adaptive ways.

In the Figure 1 the ellipses represent enzymes, proteins involved in the transmission of signals, secondary messengers, small molecules and membrane transport–related nodes. Small circles represent nodes mediating indirect regulatory interactions. The arrows represent activation, while the segments with bars indicate inhibition. The arrows in blue denote interactions extracted from species other than Arabidopsis. The nodes that are involved in the same metabolic pathway or protein complex are enclosed by rectangles and the network input is the node ABA and the output is the node ‘‘Closure’’ representing stomatal closure. Network state changes are governed by logical (Boolean) rules giving the state transition of each node according to the state of its regulators (upstream nodes). The Boolean transfer functions for each node are based on experimental evidence. The combination of the Boolean functions acting on each node in the network in time *t* is what determines the state of the node in time *t*+*1*. Here we refer to time as a sequential update of the network. See more details in section Methods.

We developed a simulation of the wild and 4 different mutant networks studied by the authors [2] and we propose and study a new mutant (see section Analysis of mutant networks). Our method of analysis consists in finding the states of the network that are most frequently visited and the identification of the variable elements because they show the activated or deactivated network sub-pathways and their biological influence on the global network behavior. The algorithm was developed using the software Mathematica 7.0 from Wolfram Research, Inc.

**Figure 2:**
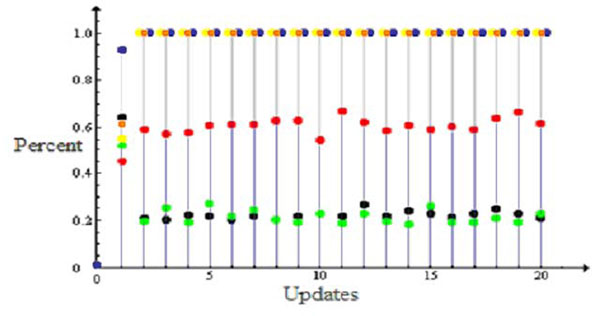
Comparison of efficiency among wild and mutant networks of affecting the closure node. Blue circles represent the wild network and yellow, red, green, orange and black represent the ABI1, S1P, pHc, NOS and PA mutants, respectively. Circles represent the percentage of simulations (300 simulations) with the closure node in state True, *i.e.*, closed stomata.

The networks have an initial period that lasts for about 3 or 4 updates where the closure node switches between open (False state) or closed (True state) depending on the initial conditions but it eventually converges to only one of this states (Fig. 2).

### The wild network Space of States (SS) Cardinality

**Figure 3:**
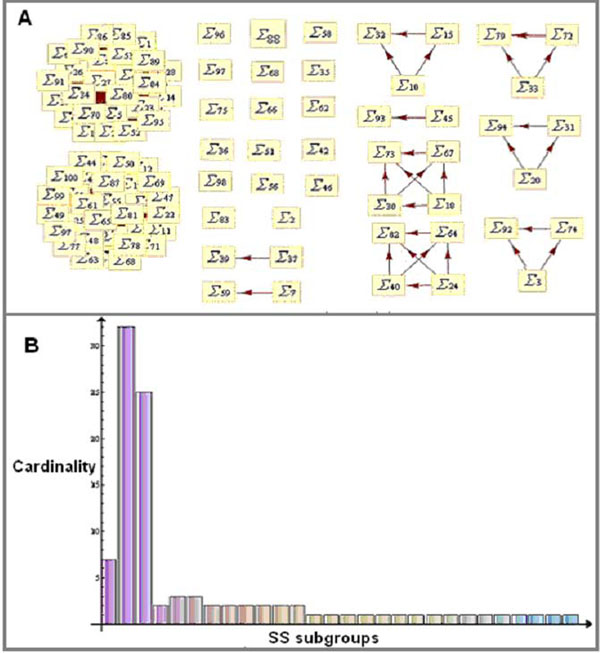
**(A)** The Space of States Cardinality. Every rectangle represents an element of the SS and the subgroups are formed by the relation of transitivity. For a given initial condition and using 100 updates of the network, we find 28 different states and the different graphs correspond to the number of times and instant of time that the specific state of the network was visited. **(B)** Histogram of the number of elements in each SS subgroup in Fig. 3(A) for the wild network. Two states are always much more frequent than the others despite the number of updates.

During reception, transduction and induction promoted by ABA in Arabidopsis several enzymes, proteins and small molecules are activated or inhibited within the cell. To study the relationship between the elements involved in signal-transduction of ABA, we use an initial random state for the network with the node ABA fixed in the True state meaning that it is being signaled. For illustration of the typical results obtained from the simulation, for a given initial condition and using 100 updates of the network, we find 28 different states in the SS that are shown in Figure 3(A). In this figure there are 28 different graphs where the elements in each one correspond to the number of times and instant of time that the specific state of the network was visited. A histogram of the number of visits of the network to each element of the groups is shown in Figure 3(B).

Depending on the number of updates and the initial condition, we obtain different cardinalities for the SS. Using 100, 200 and 300 updates and a set of 30 random initial conditions for each we obtain the average and the standard deviation of the cardinality of SS. The values obtained are shown in Table 1.

**Table 1:** Updates vs. number of states. Algorithm timings obtained with 32-bit version of Mathematica 7.0 running in a personal computer with Intel Xeon X5355 processor and 4Gb of RAM. For the simulations with 100, 200, 300 and 400 updates 30 initial conditions were used except for 500 updates where only initial condition was used given to its very long duration.

Updates	Average number of states	StandardDeviation	Average running time (s)
100	27.9	1.97135	88.3
200	33.5	1.35824	2250
300	34.4333	1.04	17900
400	36	1.01678	68000
500	36	-	253700

**Figure 4:**
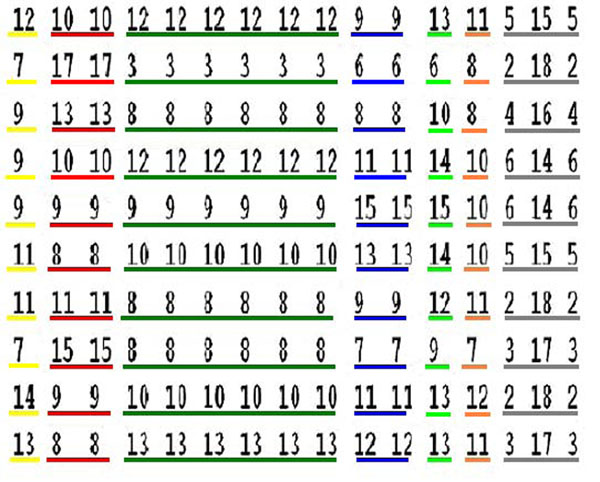
The set of 16 variable elements of the wild network (see Fig. 1) arranged in the columns and the result of 10 initial condition are shown in the rows. The numbers in the figure represent the number of times, out of 20 (updates), that a given variable element was found in the True state. Groups of elements with the same number are in the same state. The colors help to distinguish the different groups. The elements in the three last columns (KAP, KEV e Ca^2+^_c_ ) are not in the same state.

Checking the state of each node for the updates we find that only 16 out of 43 are variable elements, while the remaining 27 are frozen in True or False states. Due to the fast increasing computational time, we had to manage the number of initial conditions and updates used. The variable elements were determined using simulations with 300 different initial conditions and 10 updates, 100 initial conditions with 50 updates and 2 different initial conditions with 800 updates. In each simulation the variable and frozen elements are the same and the results show that the wild network and the mutant networks have different sets of variable elements (see Fig. 4).

**Figure 5:**
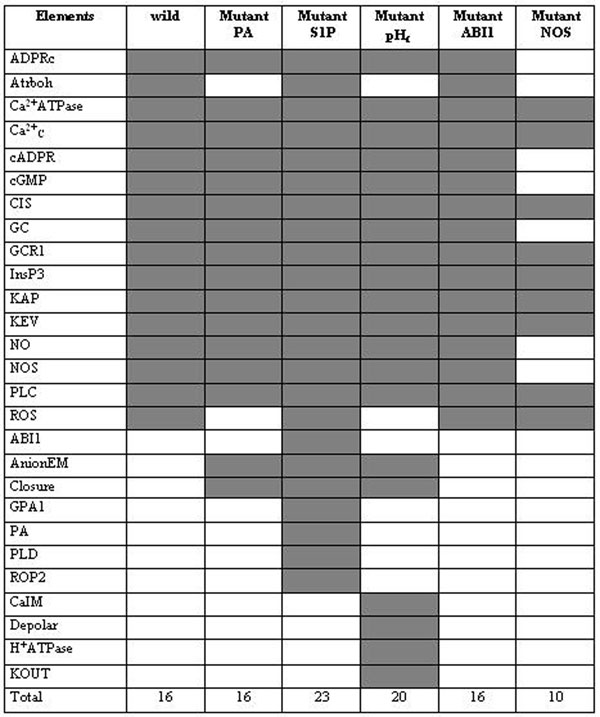
The variable elements of the wild and mutant networks are marked by gray boxes in the table.

Considering the results obtained in Fig. 1 and Fig. 4, we observe that some elements can synchronize their states as a result of the network topology and dynamics. Disregard the number of updates and the initial conditions used the variable elements are arranged in groups where all are on the same boolean state, some belong to the same metabolic pathway but other nodes have the same state due to the Boolean rules that define their states. We find from the simulations that the groups with the same color remain in the same state independent of the initial conditions used (Fig. 5).

This is illustrated in Figure 5 for simulations with 10 different initial conditions and 20 updates. In this figure we show all 16 variable elements (the respective node name is not shown and is not relevant) found which are arranged in columns and the result of each initial condition are the rows. The numbers in the figure represent the number of times, out of 20 (updates), which a given variable element was found in the True state. This explains the low cardinality of the SS, as many nodes are found in the same state, the network visits a very small portion of the 2^43^ theoretically possible states.

Figure 1 shows the nodes referred in Figure 5, with corresponding colors. The nodes that are held in a fixed state are marked with light ellipses. The elements KAP, KEV e Ca^2+^_c_ (the three last columns of Figure 5) are not in the same state, but constitute a separate group of variable elements since they are most of the time only in the True or in the False states. Particularly, the KAP and KEV are most frequently found in the False state, while Ca^2+^_c_ in the True state.

#### Analysis of the mutant networks

In their original paper Albert [2] studied the influence on the dynamics of the network arising from four experimentally studied perturbations of the network (called mutations by the authors) involving the nodes S1P, PA, ABI1 and pHc. These mutants are simulated by holding the elements S1P, PA, ABI1 and pHc in the False state.

In addition, we predict the behavior of a new *in silico* mutant using nitric oxide synthase (NOS) due to its importance to NO biosynthesis as well as being a crucial element for defining the variant dynamics of states of various other network elements as shown in Figure 1 by the group of elements highlighted in green. Nitric oxide (NO) is a key factor involved in stomatal closure in response to ABA and probably in response to other stimuli including oxidative stress induced by a range of abiotic conditions including water stress. During water stress ABA has several ameliorative functions involving NO as a key signaling intermediate including the rapid induction of stomatal closure and the activation of antioxidant defenses [3-5].

The behavior of the wild and mutant networks is shown in Figure 2. The simulations used 300 random initial conditions and lasted 20 updates each with node ABA held active implying that the hormone is being signaled by the plant.

Through our simulations we found that it is impossible to have random boolean states for the pair of nodes { AnionEm, closure }. For the mutants PA, S1P and pH_c_ , whose efficiency to set the closure node on state ON is low, this pair alternates between the states {True,True} or {False,False}. For the wild and the mutants NOS and ABI1, whose efficiency to set the closure node on state ON is high, the pair assumes only the states {True,True}. This shows that these two nodes are synchronized in all different networks and we believe that this is an essential component of the dynamics. This observation explains the difference in behavior between the wild and mutant networks and was not observed in the original paper of Albert [2]. This synchronization between closure and AnionEm nodes is experimentally supported [6]. The mutation in S1P, PA and pHc networks causes the state variation of AnionEm that interferes indirectly in the states of the KAP and KOUT nodes (see Fig. 1 and Fig. 4) making the closure node variable and consequently the stomata alternate between open and closed (Fig. 2).

The intersection set of variable elements involving the wild and the 5 mutants listed in Figure 4, are the following 8 elements:

{ Ca^2+^ATPase, Ca^2+^_c_ , CIS, GCR1, InsP3, KAP, KEV, PLC }

Is clearly seen from Figure 4 (and Fig. 2) that the mutants S1P, PA and pH_c_ make the closure node a variable element, differently from the wild and the mutants ABI1 and NOS where it gets fixed in the True state for all simulations, implying that the stomata are closed in the plant. Table 2 shows that single node mutations can affect substantially the cardinality of the networks. The comparison of the SS of the wild and mutant networks shows different cardinalities with the presence of two most frequent states for all of them (Fig. 3(B)). This seems to be a robust feature of the dynamics of these networks since this pair of most frequent states is present in the wild and mutated networks though they are not the same among the networks.

**Table 2:** Cardinality of the SS of the networks. The cardinalities were determined by taking the average of 30 simulations during 100 updates with random initial conditions.

Network type	SS Cardinalities
wild	27.9
S1P mutant	26
PA mutant	16
pH_c_ mutant	20
ABI1 mutant	28
NOS mutant	18.5

## Conclusions

In this paper we introduced a method of analysis of random boolean models of regulatory networks based on the study of the SS of the network. Its application to a modified version of the model proposed by Albert and coworkers [2] allowed us to characterize its dynamical behavior in the SS. For both, wild and mutant networks we find an identical feature: only two states are highly visited in the SS and are different for each network which seems to be a central feature of their dynamics. We also predicted an apparently key mechanism for the activation of the closure node: its synchronization with the AnionEm node (representing the efflux of anions at the plasma membrane). Comparing the dynamics of the simulated mutants with of the wild network, we find that PA, S1P and pHc mutations destabilize the synchronization of the anion and closure nodes in the True state, while for the wild and the highly efficient simulated mutants NOS and ABI1 they are fixed in this state. Experimental studies with these mutants support the behavior of the simulated networks and the involvement of the efflux of anions at the plasma membrane in the process [6-9]. Our study predicts that the involvement of the anion efflux is crucial for the plant response to ABA what remains to be confirmed by the experimental progress in the field.

## Methods

Definition: A boolean network consists in a group of *N* nodes or elements *{N_1_*, *N_2_*, *...* ,*N_N_ }* so that

σ_i_ ∈ {True, False}

is the state of the *N_i_* node. The state True or False of each node is determined by the initial conditions along with the Boolean rules that determine the state of *N_i_* determined by the state of its regulators {*N_i1_*, *N_i2_* ,*...*, *N_ik_*}.

The boolean operations are used as follows: if two or more elements can induce the activation of a node in an independent way, we combine both with the logical function OR, if two or more components cannot induce the activation in an independent way, we associate to both the logical operator AND and finally, the operator NOT will be associated to the inversion of the state of the element.

Our method of analysis consists in finding the states of the network that are most frequently visited and the identification of the variable elements since they show the activated or deactivated network sub-pathways and their biological influence on the global network behavior. As detailed below.

The algorithm describing the network logics is updated sequentially changing the states of the nodes as a result of their interactions. However, some elements with no prior information about what determine their states are updated with a random boolean state what defines a hybrid synchronous/asynchronous dynamics that is different from that used in reference [2]. To illustrate the situation of random elements consider, for instance, node GCR1 that inhibits GPA1 in Fig. 1. At each update GCR1 receives a random state. So, although the interactions in the network are deterministic the random updates of some nodes imply that the network evolves randomly in time. Denoting by

Σ_t_ ={σ_1_(t) , σ_2_(t), … , σ_k_(t)}

the state of the nodes in the update *t*, we have that(1)

represents a set of possible states of the network for *t_m_* updates for a given initial condition and defines a possible trajectory of the network in its Space of States (SS)[10]. As we have a random boolean network the orbits are not necessarily equal for the same initial condition. The cardinality of *Σ_Total_* grows with increasing number of updates and has a maximum of *Ω*=*2^N^* different elements.

To characterize a trajectory we search for equal elements in (1) for each initial condition. The search for these states allows us to establish a relation with the frequency that the state is visited. To find equal states we use the Hamming distance *D*: If *Σ_t_*, *Σ_t’_* are elements of *Σ_Total_* in updates *t* and *t’* and *σ_i_*(*t*) = *σ_i_*(*t’*) for all i, then *D*(*Σ_t_*, *Σ_t’_*) =*0*. After identification of the states in the updates we count the number of times each one is visited.

Another important feature of the dynamics of a boolean network is the general behavior of its nodes. Often, a network breaks apart in two groups of nodes for all initial conditions: in the first group the nodes are always in a frozen state and in the second the nodes are always in a variable state [11]. This feature determines the cardinality of *Σ_Total_* because if the fraction of frozen states is high, the cardinality will be low and vice-versa. We identify all frozen and variable elements of the network for analysis.

## Authors’ contributions

C. A. Bugs developed the Mathematica code of the simulations, generated the results and wrote the paper. J. C. M. Mombach designed the investigation and wrote the paper. G. R. Librelotto helped with the analysis of the results. All authors read and approved the final manuscript.

## Competing interests

The authors declare that they have no competing interests.
